# α‐Glyceryl‐phosphoryl‐ethanolamine protects human hippocampal neurons from ageing‐induced cellular alterations

**DOI:** 10.1111/ejn.15783

**Published:** 2022-08-12

**Authors:** Elisa Zappelli, Simona Daniele, Lorenzo Ceccarelli, Matteo Vergassola, Lorella Ragni, Giorgina Mangano, Claudia Martini

**Affiliations:** ^1^ Department of Pharmacy University of Pisa Pisa Italy; ^2^ Global R&D PLCM—Angelini Pharma S.p.A. Ancona Italy; ^3^ Scientific Consultant Rome Italy

**Keywords:** ageing, neuronal metabolism, neuronal plasticity, phospholipid precursor, α‐glycerylphosphorylethanolamine

## Abstract

Brain ageing has been related to a decrease in cellular metabolism, to an accumulation of misfolded proteins and to an alteration of the lipid membrane composition. These alterations act as contributive aspects of age‐related memory decline by reducing membrane excitability and neurotransmitter release. In this sense, precursors of phospholipids (PLs) can restore the physiological composition of cellular membranes and ameliorate the cellular defects associated with brain ageing. In particular, phosphatidylcholine (PC) and phosphatidylethanolamine (PE) have been shown to restore mitochondrial function, reduce the accumulation of amyloid beta (Aβ) and, at the same time, provide the amount of acetylcholine needed to reduce memory deficit. Among PL precursors, alpha‐glycerylphosphorylethanolamine (GPE) has shown to protect astrocytes from Aβ injuries and to slow‐down ageing of human neural stem cells. GPE has been evaluated in aged human hippocampal neurons, which are implicated in learning and memory, and constitute a good in vitro model to investigate the beneficial properties of GPE. In order to mimic cellular ageing, the cells have been maintained 21 days in vitro and challenged with GPE. Results of the present paper showed GPE ability to increase PE and PC content, glucose uptake and the activity of the chain respiratory complex I and of the GSK‐3β pathway. Moreover, the nootropic compound showed an increase in the transcriptional/protein levels of neurotrophic and well‐being related genes. Finally, GPE counteracted the accumulation of ageing‐related misfolded proteins (a‐synuclein and tau). Overall, our data underline promising effects of GPE in counteracting cellular alterations related to brain ageing and cognitive decline.

## Graphical abstract

α‐Glycerylphosphorylethanolamine (GPE) counteracted the decrease of membrane phospholipids induced by cellular ageing in human hippocampal neurons. GPE increased glucose uptake, the activity of the chain respiratory complex I, and of the GSK‐3β pathway. Moreover, the nootropic drug showed an increase in the transcriptional/protein levels of neurotrophic and well‐being related genes and counteracted the accumulation of ageing‐related misfolded proteins (α‐synuclein and tau).

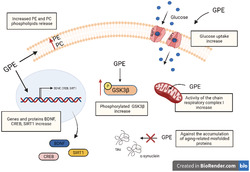

**Abbreviations**
2DG: 2‐deoxyglucose
2DG6P: 2‐deoxyglucose‐6‐phosphate
ANOVA: analysis of variance
Aβ: amyloid beta protein
BDNF: brain‐derived neurotrophic factor
BSA: bovine serum albumin
CREB: cAMP response element‐binding protein
DIV: days in vitro
DSB: double strand break
ELISA: enzyme‐linked immunosorbent assay
G6PD: glucose‐6‐phosphate dehydrogenase
GPE: alpha‐glycerylphosphorylethanolamine
GSK3: glycogen synthase kinase 3
IGF‐1: insulin‐like growth factor 1
KO: knock‐out
LTD: long‐term depression
LTP: long‐term potentiation
PBS: phosphate‐buffered saline
PBS‐T: phosphate buffered saline containing .01% Tween 20
PC: phosphatidylcholine
PE: phosphatidylethanolamine
PI: phosphatidylinositol
PL: phospholipid
SIRT1: sirtuin
TMB: enzyme substrate 3,3′,5,5′‐tetramethylbenzidine

## 1 INTRODUCTION

Ageing is a multifactorial process characterised by the decline of biological functions, memory loss and attention. In particular, several cellular alterations have been involved in the ageing process, including metabolism and mitochondrial dysfunctions, telomere shortening, oxidative stress and systemic inflammation (López‐Otín et al., 2013). During ageing, neuronal metabolism decreases with the consequence of reduction in uptake of glucose, in adenosine triphosphate (ATP) and glucose transporter 1 (GLUT1) receptor levels (Błaszczyk, 2020), in electron transport chain activity, in particular mitochondrial complex I (Pollard et al., 2016), and in aerobic glycolysis (Goyal et al., 2017).

In addition, ageing has been related to the general dysfunctions of neuronal membranes and a decline synaptic plasticity, which are responsible of cholinergic neurons disruption and the consequent deficiency in cholinergic system (Maurer & Williams, 2017; Schliebs & Arendt, 2011). The reduction in neuronal membrane functionality and the related inhibition in synaptic plasticity processes have been supposed to be the consequence of modifications in biophysical properties and in the qualitative/quantitative phospholipid (PL) composition of the plasma membrane (Egawa et al., 2016; García‐Morales et al., 2015; Ovsepian et al., 2012).

The beneficial effects of PL have been widely described in brain ageing. For instance, phosphatidylcholine (PC) administration has been shown to increase long‐term declarative memory (Ladd et al., 1993) and to act as an acetylcholine reserve (Blusztajn et al., 1987). Moreover, phosphatidylethanolamine (PE) has been demonstrated to be essential for respiratory complexes and for regulation of autophagy phenomena (Patel & Witt, 2017). Additionally, it has been shown that low levels of PE can lead to accumulation of α‐synuclein (Wang et al., 2014) and that cellular levels of PE regulate the accumulation of amyloid beta (Aβ) to the pivotal component of amyloid plaques typical of Alzheimer's disease (Nesic et al., 2012). Therefore, as PC and PE levels have been shown to decrease with age (Johnson & Stolzing, 2019), it is not surprising that changes in the brain cells composition of PC and PE have been correlated with the onset of neurodegenerative diseases (Ellison et al., 1987; Prasad et al., 1998; Söderberg et al., 1991) as well as an increase in polyunsaturated fatty acids is able to restore neuronal membranes (Yehuda et al., 2002) and to protect against oxidative stress (Pamplona et al., 2002).

α‐Glycerylphosphorylethanolamine (GPE) is a precursor of the main constituents of the cellular membrane, PE and PC (Biggio et al., 2021; Bisaglia et al., 2004; Daniele, Da Pozzo, et al., 2016). GPE has shown to ameliorate reactive gliosis caused by Aβ (Bisaglia et al., 2004) and to exert beneficial effects in aged human neural stem cells, as well as in human hippocampal neurons (Daniele et al., 2020). However, the effect of GPE on glucose metabolism, mitochondrial activity, membrane plasticity and neurodegeneration‐associated protein levels, in a model of aged neurons, has not been explored yet. The aim of the present study was to investigate GPE metabolism and neuronal plasticity effects in aged human hippocampal neurons. These neurons possess an efficient cholinergic neurotransmission (Orta‐Salazar et al., 2014) and are involved in memory, cognition and learning processes (Wood et al., 2000), and can be employed as a good in vitro model to test GPE effects in an aged brain. In parallel experiments, choline alphoscerate (α‐glycerylphosphorylcholine (Di Salvo, 2021) was used to further explore the putative neuroprotective effects of membrane precursors. This reference compound was chosen as choline/acetylcholine precursor that is used for the improvement of cognitive dysfunction in patients with dementia of neurodegenerative and vascular origin (Di Salvo, 2021; Parnetti et al., 2001; Perri et al., 1991).

## 2 MATERIALS AND METHODS

### 2.1 Cell culture and ageing model of hippocampal neurons

Human or rat hippocampal cells were purchased from Innoprot (Innoprot, Bizkaia, Spain) and maintained in culture as reported (Ray et al., 2014). Those cells have been characterised by immunofluorescent method and have been shown to express neurofilament, MAP‐2 and beta‐tubulin III proteins (https://innoprot.com/product/human-hippocampal-neurons/).

The cells were cultured for 21 DIV (days in vitro) in order to mimic a state of neuronal ageing, as reported in the literature and verified in previous experiments (Calvo‐Rodríguez et al., 2017; Daniele et al., 2020; Daniele, Da Pozzo, et al., 2016). Human hippocampal neurons at 3 DIV were used as “young neurons”.

In order to confirm the induction of cellular ageing, the expression of key proteins related with the ageing process (i.e., p53, p21 and glucose‐6‐phosphate dehydrogenase, G6PD) was verified at different times of cell culture. Briefly, hippocampal cells (at 3, 10 and 21 DIV) were fixed with 4% formaldehyde, and protein levels were determined by enzyme‐linked immunosorbent assay (ELISA) assays, as previously reported (Daniele et al., 2019; Daniele, Barresi, et al., 2016; Fumagalli et al., 2015; Giacomelli et al., 2015). In particular, the cells were washed three times with wash buffer (.1% Triton X‐100 in phosphate‐buffered saline [PBS]) and 100 μl of quenching buffer (1% H_2_O_2_; .1% sodium azide in wash buffer) was added for other 20 min. The cells were washed with PBS twice, and then 100 μl of blocking solution (1% bovine serum albumin [BSA]; .1% Triton X‐100 in PBS) was added for 60 min. After blocking, cells were washed three times with wash buffer and incubated with the following specific primary antibodies: p21, sc‐6246, G6PD, sc‐373886 (Santa Cruz Biotechnology, DBA Italia, Milan, Italy), p53, 70R‐31561 (Fitzgerald, Gentaur SRL, Bergamo, Italy). Then, a secondary HRP‐conjugated antibody and developing solution were employed. Blanks were obtained by treating cells in the absence of the primary antibody. The relative number of cells in each well was then determined using Crystal Violet solution. The results were calculated by subtracting the mean background from the values obtained from each test condition; values were normalised to the number of cells in each well and were expressed as the percentage of untreated cells (basal).

### 2.2 Pharmacological treatments

During these 21 days, the cells were treated with GPE at three different concentrations (5, 50 and 500 μM), selected on the basis of previous in vitro experiments (Daniele et al., 2020). In parallel experiments, choline alphoscerate was tested at the highest concentration**s** (i.e., 50 and 500 μM) to compare its maximal effects with those obtained with GPE. GPE was provided by Angelini S.p.A. Stock solutions of differing GPE concentrations were created by dilution with PBS. All other reagents were of the highest commercially available grade and obtained from standard commercial source.

### 2.3 Quantification of PC and PE

Hippocampal neurons were tretaed with medium alone (control cells) or 500 μM GPE for 21 DIV. Following incubation, PLs were quantified by commercial fluorometric assays (Daniele et al., 2020) in which enzyme‐coupled reactions were used to hydrolyse PC (ab83377 Abcam, Cambridge, UK) and PE (#K499‐100 Biovision, Biovision Incorporated, Milpitas, CA, USA). The data are expressed as nanomoles of PC or PE/sample and normalised for the number of cells in each well.

### 2.4 Quantification of glucose uptake

Hippocampal cells (5000 cells/well) were challenged with medium (control cells) or GPE at three different concentrations (5, 50 and 500 μM) or with choline alphoscerate (500 μM) for 21 DIV. Then, glucose uptake was determined by a fluorometric method based on the detection of 2‐deoxyglucose‐6‐phosphate (2DG6P). Briefly, 2‐deoxyglucose (2DG) is added to cells, and after a brief period of incubation, an acid detergent solution (Stop Buffer) is added to lyse cells, terminate uptake, and destroy any NADPH within the cells. A high‐pH buffer solution was then added to neutralise the acid. A Detection Reagent containing glucose‐6‐phosphate dehydrogenase (G6PDH), NADP+, Reductase, Ultra‐Glo™ Recombinant Luciferase and pro‐luciferin substrate is added to the sample wells. G6PDH oxidises 2DG6P to 6‐phosphodeoxygluconate and simultaneously reduces NADP+ to NADPH. The reductase uses NADPH to convert the pro‐luciferin to luciferin, which is then used by Ultra‐Glo™ Recombinant Luciferase to produce a luminescent signal that is proportional to the concentration of 2DG6P. The data were reported as the fold change from control cells set to 100%.

### 2.5 Quantification of IGF‐1 levels

Human neurons (5000 cells/well) were challenged with medium (control cells) or GPE at three different concentrations (5, 50 and 500 μM) or with choline alphoscerate (500 μM) for 21 DIV. Then, IGF1 levels were determined by an in vitro linked immunosorbent assay (ab100545 Human IGF1 ELISA Kit, Abcam) according to manufacturer's instruction. The data were reported as the fold change from control cells set to 100%.

### 2.6 Quantification of the chain respiratory complex I activity

Human neurons (5000 cells/well) were treated with medium alone (control cells) or GPE at three different concentrations (5, 50 and 500 μM) or with choline alphoscerate (500 μM) for 21 DIV. Then, chain respiratory complex I activity was determined by an in vitro ELISA (MitoTox Complex I OXPHOS activity assay, Abcam ab109903). Briefly, 50 μl of the diluted mitochondria to each well was added and incubated for 2 h at room temperature. After incubation time, 40 μl of PLs to each well was added and incubated for 45 min at room temperature. In fine, Complex I Activity solution was added for 1 h. The measure OD340 at 1‐min intervals for 2 h at 30°C was performed. The data were reported as the fold change from control cells set to 100%.

### 2.7 RNA extraction and real‐time PCR analysis

Hippocampal neurons were seeded in six‐multiwell plates (1 × 10^4^ cells/well) and incubated with medium (control cells) or GPE at three different concentrations (5, 50 and 500 μM) and with choline alphoscerate (500 μM) for 21 days (21 DIV in vitro). Then, total RNA was extracted from the collected cells using Rneasy® Mini Kit (Qiagen, Hilden, Germany). cDNA was obtained using i‐Script cDNA synthesis kit (BioRad, Hercules, USA) from 500 ng RNA. Primers were designed to exclude genomic DNA from products (see Table 1). RT‐PCR reactions were set up as reported (Daniele et al., 2015).

**TABLE 1:** Sequences and annealing temperature of the primers employed in PCR reactions

Gene	Primer nucleotide sequences	Product size (base pairs)	Annealing temperature
BDNF	FOR: 5′‐TACATTTGTATGTTGTGAAGATGTTT‐3′ REV: 5′‐CCTCTTTTCAGAAAAATTCAGGA‐3′	131	56°
SIRT1	FOR: 5′‐CCTGGACAATTCCAGCCATC‐3’ REV: 5′‐TTCATGATAGCAAGCGGTTCAT‐3’	272	66°
CREB	FOR: 5′‐AAGCTGAAAGTCAACAAATGACA‐3′ REV: 5′‐CCTCTTTTCAGAAAAATTCAGGA‐3’	240	52°
βactin	FOR: 5′‐GCACTCTTCCAGCCTTCCTTCC‐3′ REV‐5′‐GAGCCGCCGATCCACACG‐3′	254	55°

### 2.8 Quantification of BDNF, SIRT1 and CREB levels

Hippocampal cells were challenged with medium (control cells) or GPE for 21 DIV. Then, cells were fixed with 4% formaldehyde, and protein levels were determined by ELISA assays, (Daniele et al., 2019; Daniele, Barresi, et al., 2016; Fumagalli et al., 2015; Giacomelli et al., 2015), as reported above.

### 2.9 Quantification of glycogen synthase kinase 3 (GSK3) levels

Human cells were challenged with medium (control cells) or GPE at three different concentrations (5, 50 and 500 μM) and with choline alphoscerate (500 μM) for 21 DIV. Then, cells were fixed with 4% formaldehyde, and GSK3β levels (total and phosphorylated) were determined by ELISA assays, as described above. The specific primary antibodies were anti‐GSK3, sc‐7383 and p‐GSK‐3β, sc‐373800, Santa Cruz Biotechnology, DBA Italia, Milan, Italy).

### 2.10 Quantification of total α‐synuclein, total tau and phospho‐tau levels

Hippocampal cells were challenged with medium (control cells) or GPE at three different concentrations (5, 50 and 500 μM) and with choline alphoscerate (500 μM) for 21 days (21 DIV in vitro). Following incubation, the cells were collected and α‐synuclein and tau levels were quantified by an in vitro linked immunosorbent assay (Daniele et al., 2018). Briefly, wells were pre‐coated overnight at 4°C with an anti‐α‐syn (NBP2‐15365, Novus Biological, Bio‐Techne SRL, Milan, Italy) or anti‐tau (sc‐32274, Santa Cruz, DBA Italia, Milan, Italy) antibody, and non‐specific sites were blocked using BSA for 1 h at 37°C. Lysed cells were captured on wells for 2 h at 25°C. Purified recombinant protein standards of α‐synuclein and tau were assayed in parallel with human samples to generate a standard curve. After washing, the samples were probed with a mouse monoclonal antibody to α‐syn (sc‐12767, Santa Cruz) or to tau (ab109392, abcam, Prodotti Gianni, Milan, Italy, for total tau detection) or to phosho‐tau (70R‐32555, Fitzgerald Industries International, DBA Italia, Milan, Italy) (Piccarducci et al., 2019). Later, the samples were challenged with an anti‐mouse‐HRP or anti‐rabbit‐HRP antibody. The wells were then washed four times with PBS containing .01% Tween 20 (PBS‐T), before adding the enzyme substrate 3,3′,5,5′‐tetramethylbenzidine (TMB, Life Technologies, Monza, Italy) and leaving the colour to develop for 30 min at room temperature. Absorbance values were measured at 450 nm. The data were reported as the fold change from control cells set to 100%.

### 2.11 Quantification of oligomeric α‐synuclein

Oligomeric α‐syn levels in hippocampal cells were determined by a specific immunoenzymatic assay (Daniele et al., 2018; El‐Agnaf et al., 2006). The plate was coated overnight at room temperature with the mouse monoclonal α‐syn 211 antibody (Santa Cruz, sc‐12767, DBA Italia, Milan, Italy) and then incubated for 2 h with cell samples. α‐syn oligomers were quantified sing a specific α‐syn biotinylated antibody (Daniele et al., 2018; El‐Agnaf et al., 2006). Streptavidin‐horseradish peroxidase conjugate antibody (1:1000, GE Healthcare Italia, Milan, Italy) was used for antigen detection. After three washes with PBS‐T, TMB was added in each well, as reported above.

### 2.12 Statistical analysis

The data analysis was performed using one‐way analysis of variance with Bonferroni's corrected *t* tests for post hoc pair‐wise comparisons. *p* < 0.05 was considered statistically significant.

## 3 RESULTS

### 3.1 Setup of aged hippocampal neurons

**FIGURE 1:**
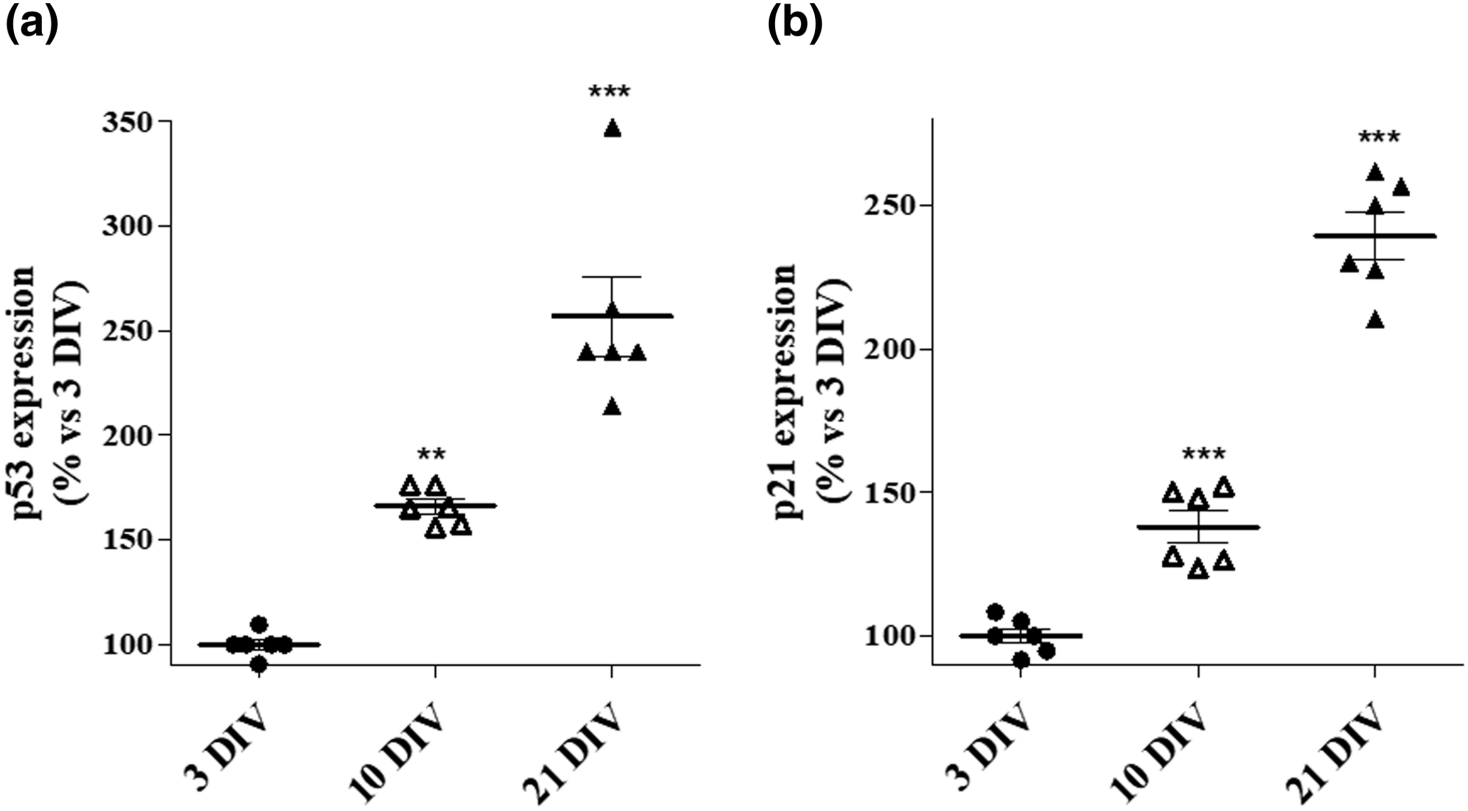
Evaluation of ageing cellular markers. Hippocampal cells were cultured for 3, 10 and 21 days in vitro (DIV). Then, cells were fixed, and the expression of (a) p53 and (b) p21 was quantified by specific immunoenzymatic assays. The data are expressed as percentage of expression versus 3 DIV, set to 100%. ** *p* < 0.01, ***p < 0.001 versus 3 DIV

Hippocampal cells have been characterised by immunofluorescent method and have been shown to express neurofilament, MAP‐2 and beta‐tubulin III proteins (https://innoprot.com/product/human-hippocampal-neurons/). As a first step, human hippocampal neurons were maintained in culture for 21 DIV (days in vitro) in order to mimic a state of neuronal ageing, as reported in the literature and verified in previous experiments (Calvo‐Rodríguez et al., 2017; Daniele et al., 2020). To further confirm the induction of cellular ageing, the expression of key proteins related with the ageing process (i.e., p53 and p21) was verified at different times of cell culture. Cultured primary hippocampus neurons at 3 DIV exhibited a significantly lower expression of p53 and p21 with respect to 10 DIV and 21 DIV (Figure 1a and b). Thus, the protein expression of p53 and p21 increased during neuronal culture in vitro, reaching their highest pick at 21 DIV. These results confirm a significant cellular senescence at 21 DIV.

### 3.2 Release of PLs in human hippocampal neurons

**FIGURE 2:**
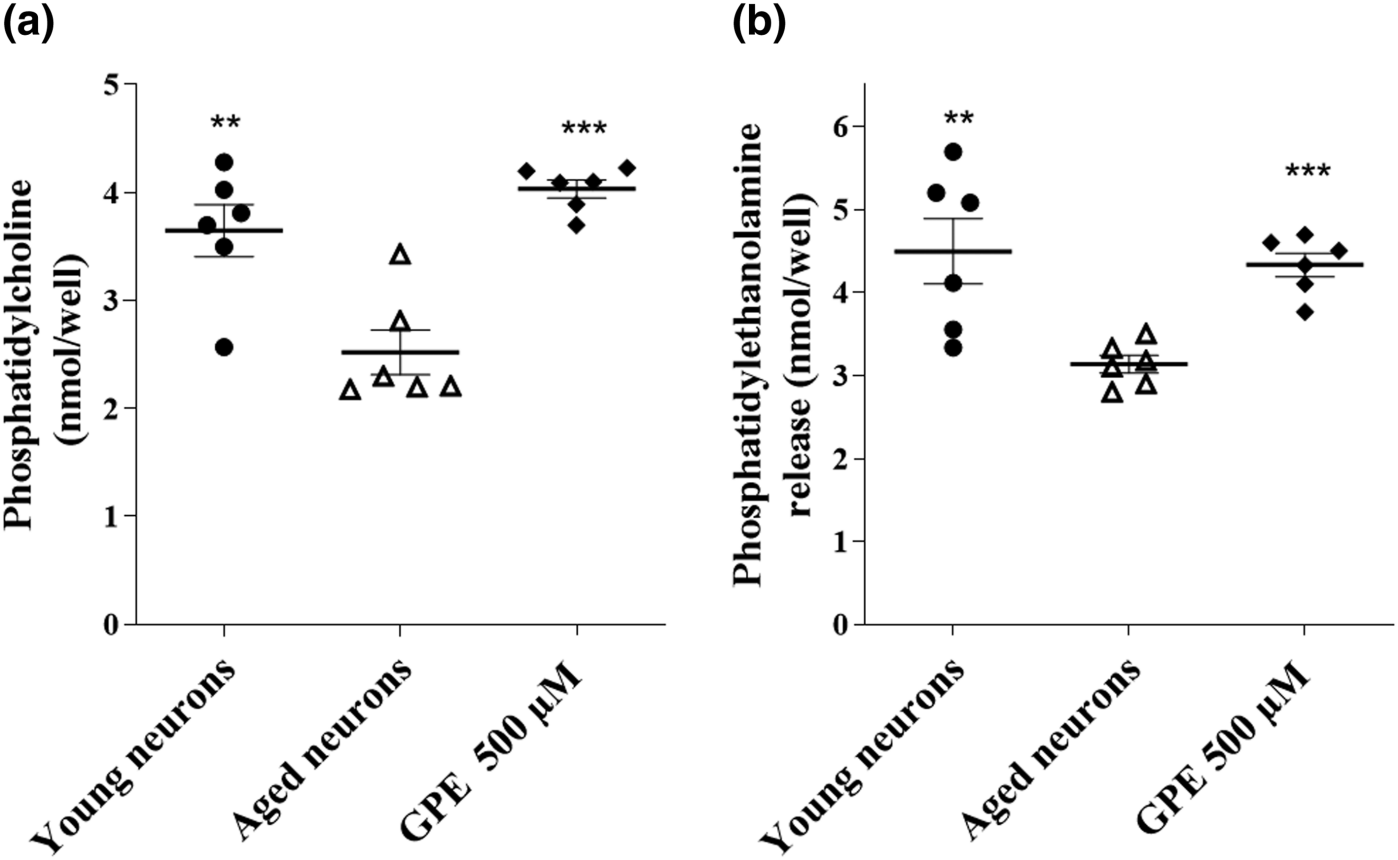
Evaluation of phospholipids content in human hippocampal cells. After treatment, the release of (a) PC and (b) PE was quantified by commercial fluorometric assays, as described in section 2. The data are expressed as nanomole of PC or PE detected in each well, and are the mean SEM of three different experiments, each performed in duplicate. ** *p* < 0.01, ***p < 0.001 versus aged cells

During the induction of cellular ageing in vitro, hippocampal neurons were challenged every 3 days with GPE at three different concentrations (5, 50 and 500 μM), as reported in the previous work (Daniele et al., 2020), or with choline alphoscerate (500 μM). As a first step, the content of the main PLs, PC and PE, was verified in the aged cellular model. Hippocampal cells at 21 DIV displayed a significant decrease in the amount of PLs (Figure 2a and b). Challenging human hippocampal neurons with GPE for 21 days significantly counteracted the reduction in the PC (Figure 2a) and PE Figure 2b) content. These data confirm that GPE promote PLs biosynthesis and are consistent with our previous results obtained in previous reports (Daniele et al., 2020; Daniele, Da Pozzo, et al., 2016).

### 3.3 Evaluation of glucose uptake levels

**FIGURE 3:**
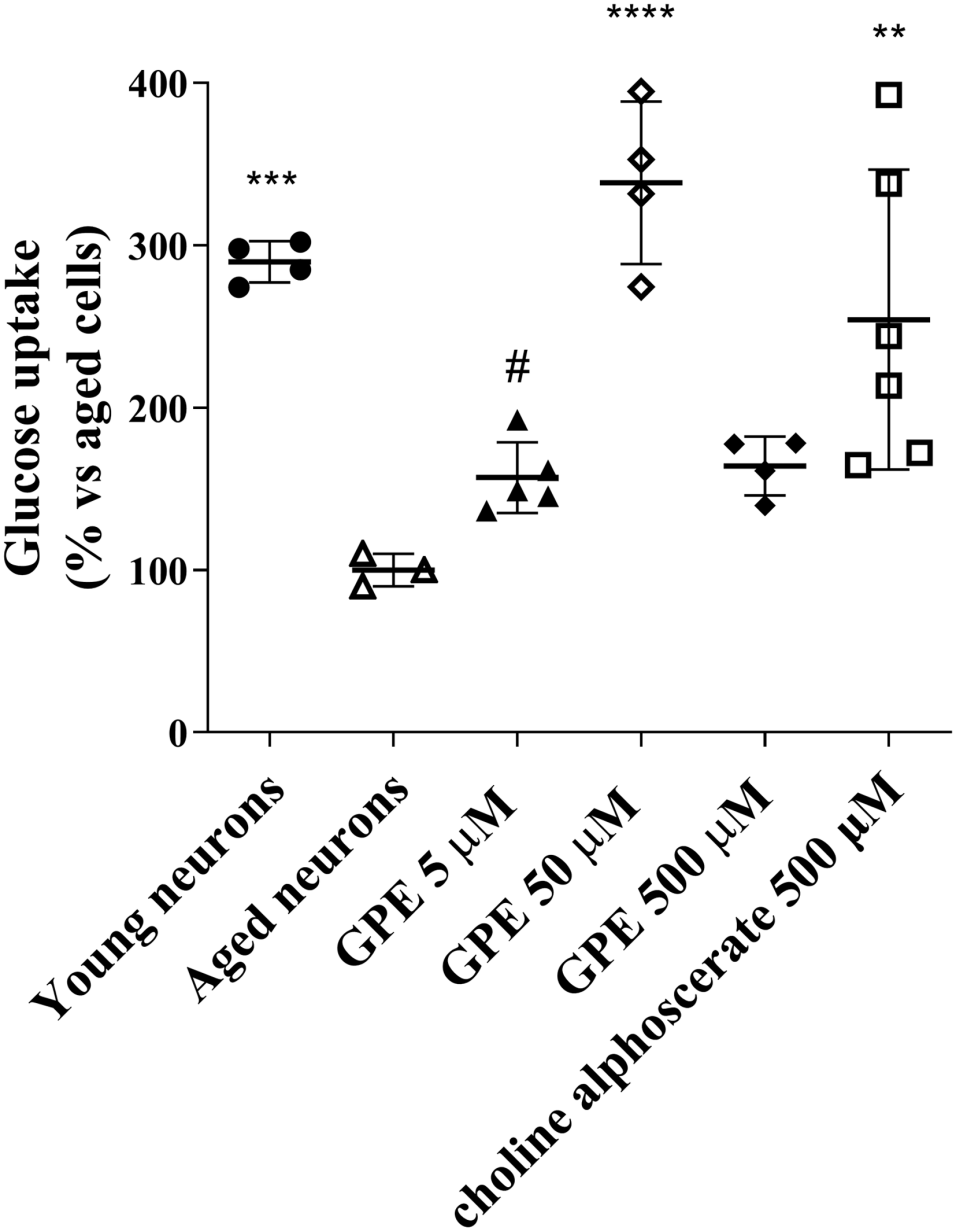
Evaluation of glucose uptake capacity. Aged human hippocampal neurons were incubated with culture medium only (aged cells) or GPE at three different concentrations (5, 50 and 500 μM), or choline alphoscerate (500 μM) for 21 days. Hippocampal neurons at 3 DIV were used as “young neurons”. At the end of the treatments, the cells were collected, and the rate of glucose uptake was determined by a luminescent assay (Glucose Uptake‐Glo ™ Assay, Promega). The data are expressed as a percentage with respect to the control (set at 100%) and represent the mean ± SEM of three separate experiments, executed in triplicate. **p < 0.01, ***p < 0.001, ****p < 0.0001 versus aged cells; #*p* < 0.05 versus choline alphoscerate

Brain functions such as thinking, memory and learning are closely linked to glucose levels and how efficiently the brain uses this fuel source (Dwyer, 2002). Unfortunately, neuronal metabolism is compromised during ageing because of a series of events including a reduction in glucose uptake (Castelli et al., 2019). In this sense, as a first step, we aim to understand if GPE can counteract the reduction of glucose uptake in aged hippocampal neurons. As expected, human neurons at 3 DIV showed significantly higher levels of glucose uptake (Figure 3) with respect to aged neurons. Challenging cells with GPE for 21 days induced an increase in glucose uptake capacity compared to control cells, which was significant at 50 μM (Figure 3); in parallel experiments, choline alphoscerate induced a significant glucose uptake but lower than that obtained with GPE 50 μM (Figure 3). Overall, these data demonstrate that GPE is able to increase glucose uptake at lower concentrations than choline alphoscerate.

### 3.4 Evaluation of IGF‐1 levels

**FIGURE 4:**
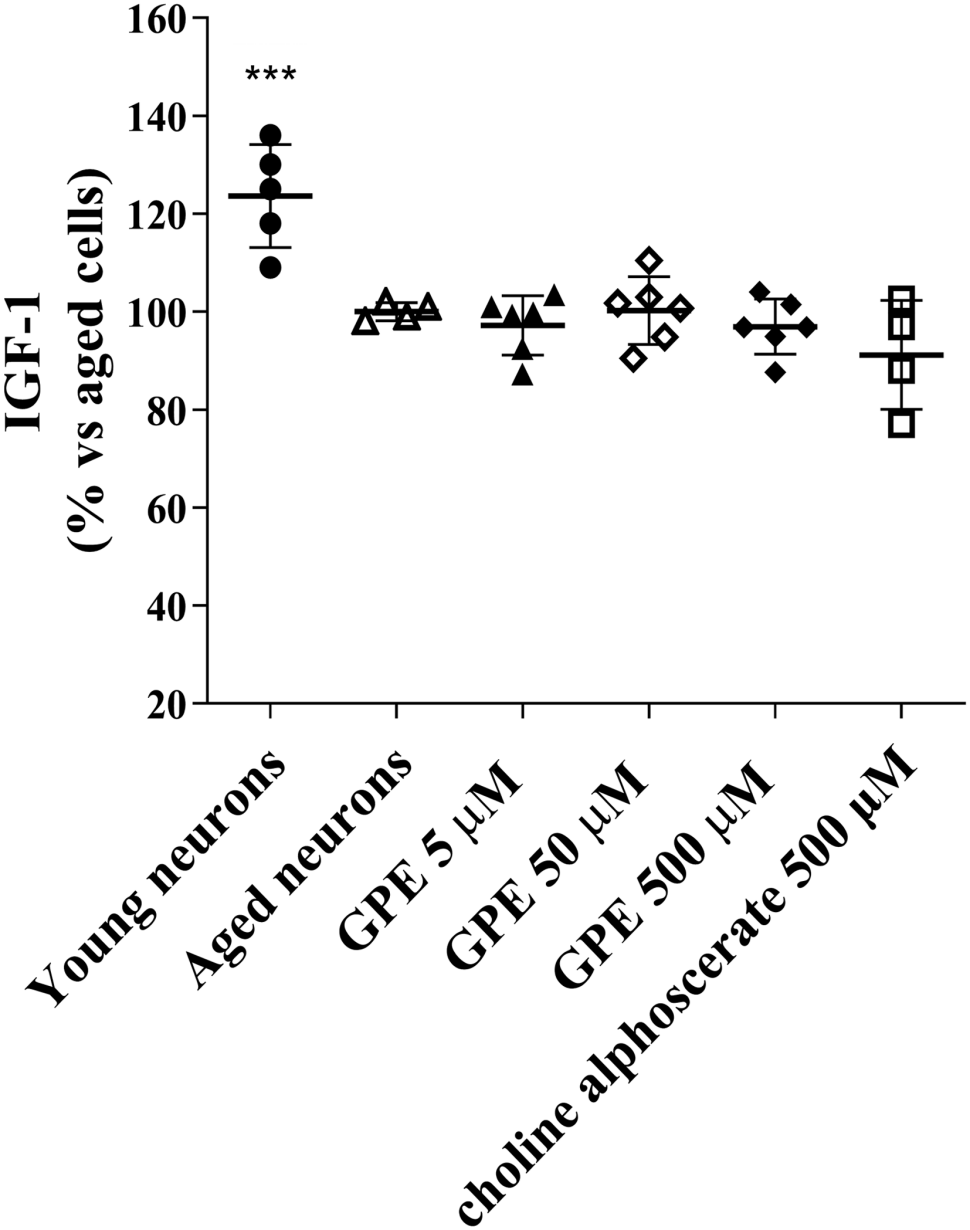
Evaluation of IGF‐1 release. Aged human hippocampal neurons were incubated with culture medium only (control cells) or GPE at three different concentrations (5, 50 and 500 μM), or choline alphoscerate (500 μM) for 21 days. The release of IGF1 into the culture medium was determined by an immunoenzymatic assay (human IGF1 ELISA kit, ab100545). The data are expressed as a percentage with respect to the control (set at 100%) and represent the mean ± SEM of three separate experiments, executed in triplicate. ***p < 0.001 versus aged cells

Although insulin‐like growth factor 1 (IGF1) signalling pathway has emerged as a major regulator of the ageing process, many controversial opinions persist (Gubbi et al., 2018). To investigate GPE effect on IGF‐1 hormone levels, aged hippocampal cells were treated with different GPE concentrations or 500 μM choline alphoscerate for 21 days, and IGF‐1 released in the culture medium was assessed by an immunoenzymatic assay. The results show that neither GPE nor choline alphoscerate induced a significant change in IGF‐1 levels (Figure 4). Of note, human neurons at 3 DIV showed significantly higher IGF‐1 levels (Figure 4) with respect to aged neurons.

### 3.5 Evaluation of oxidative phosphorylation

**FIGURE 5:**
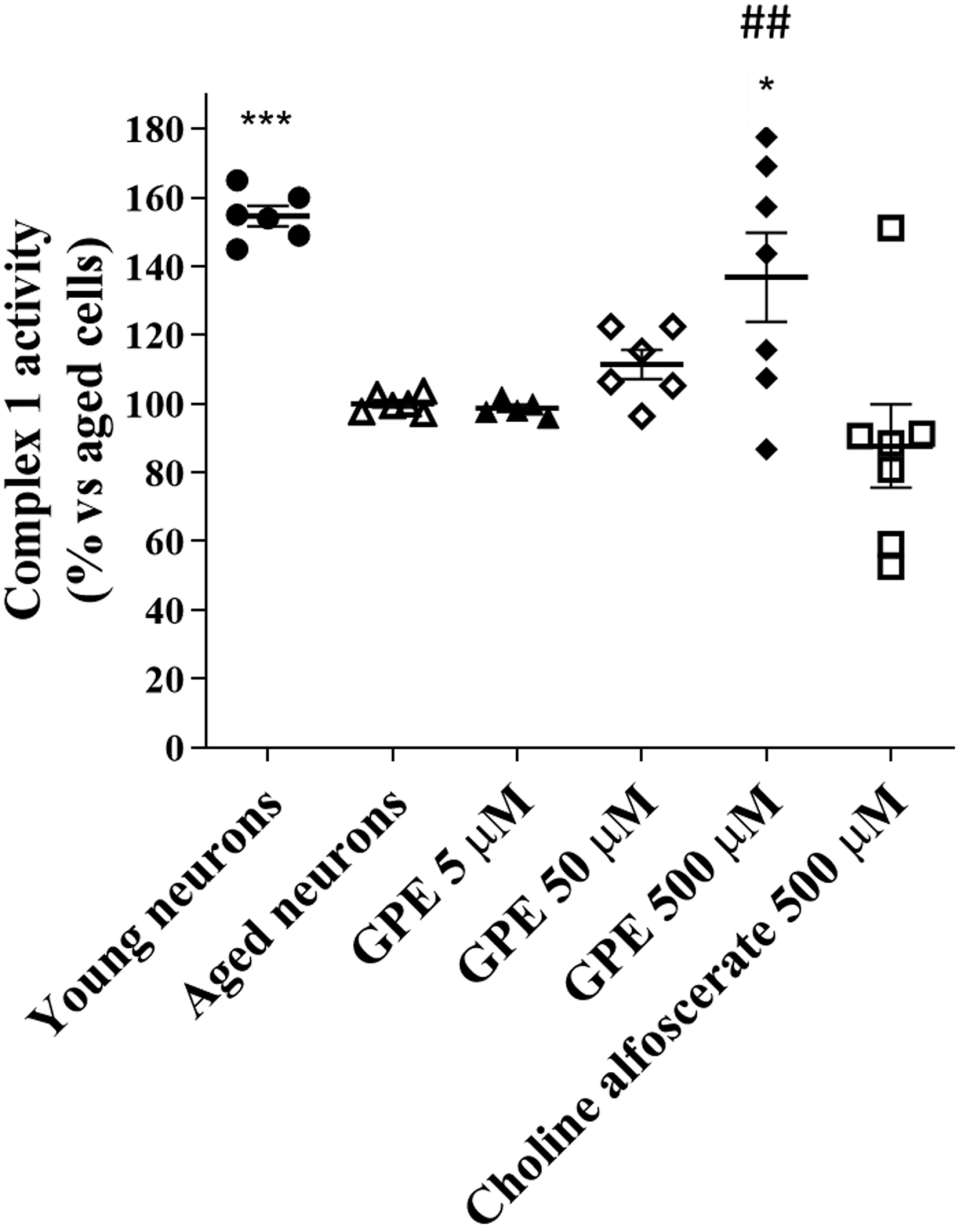
Evaluation of the activity of complex I of the respiratory chain. Aged human hippocampal neurons were incubated with culture medium only (control cells) or GPE (5, 50 and 500 μM), or choline alphoscerate (500 μM) for 21 days. At the end of the treatments, the mitochondria were isolated by centrifugation, and the activity of complex I of the respiratory chain was evaluated by an in vitro ELISA (MitoTox Complex I OXPHOS activity assay, Abcam ab109903). The data are expressed as a percentage with respect to the control (set at 100%) and represent the mean ± SEM of three separate experiments, executed in triplicate. *p < 0.05, ***p < 0.001 versus aged cells; ## *p* < 0.01 versus choline alphoscerate

Activity of complex I of the electron transport chain has been shown to decrease during normal ageing and in neurodegenerative disease (Pollard et al., 2016). Consistently, human neurons at 3 DIV (“young neurons”) showed significantly higher oxidative phosphorylation (Figure 5) with respect to aged neurons. Having demonstrated that GPE is able to increase glucose uptake, its effect on mitochondrial activity was investigated. GPE significantly increased the respiratory chain complex I activity at 500 μM concentration (Figure 5). In parallel experiments, choline alphoscerate did not show any effect on complex I activity. Globally, these results confirm the GPE positive effect at the mitochondrial level.

### 3.6 Evaluation of induction of genes and proteins related to neuronal plasticity

**FIGURE 6:**
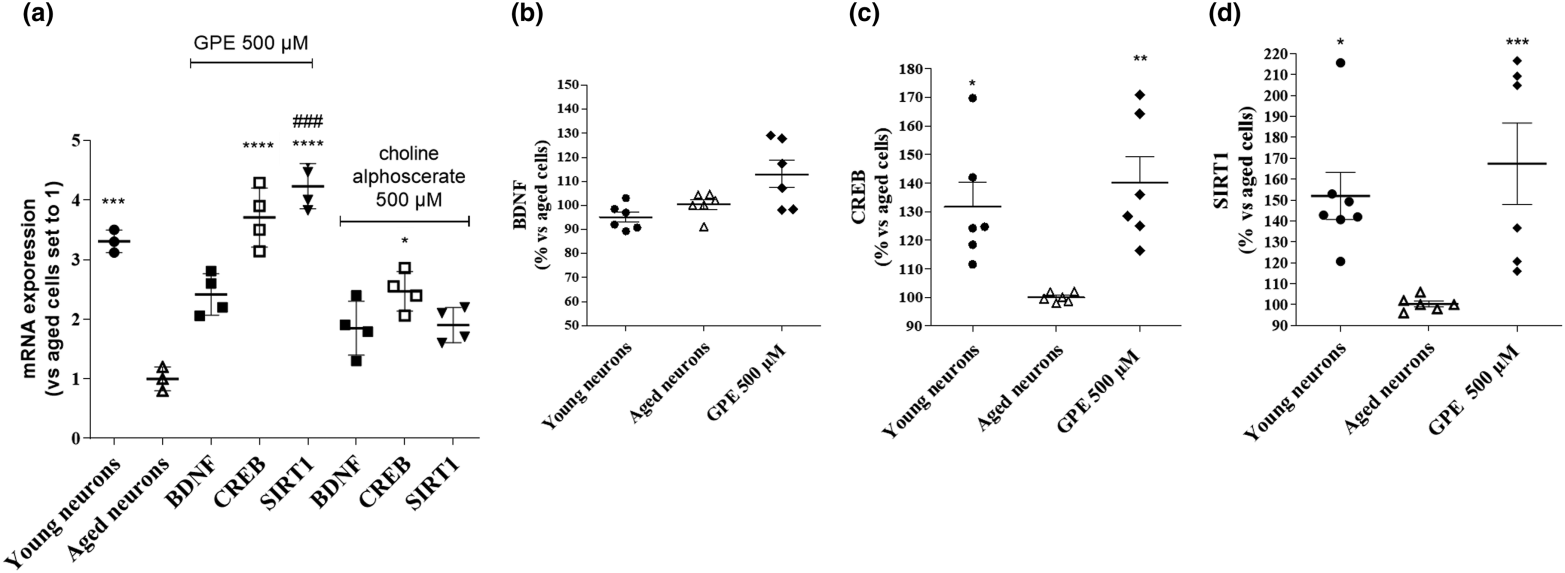
Evaluation of the induction of plasticity membrane‐correlated genes and proteins. (a–d) Aged hippocampal neurons were incubated with culture medium only (control cells) or GPE (500 μM), or choline alphoscerate (500 μM) for 21 days. (a) At the end of the treatments, the mRNA was extracted and the CREB, BDNF and SIRT‐1 mRNA levels were quantified by real‐time analysis RT‐PCR. The data are expressed as fold of changes compared to control cells (set to 1) and represent the mean ± SEM of three separate experiments, executed in duplicate. Hippocampal cells were treated as in Figure 6a. Then, cells were fixed, and the expression of (b) BDNF, (c) CREB and (d) SIRT1 were quantified by specific immunoenzymatic assays. The data are expressed as percentage of expression in aged neurons, set to 100%. **p < 0.05, **p < 0.01, ***p < 0.001 versus aged cells; ###*p* < 0.001 versus choline alphoscerate

Ageing is associated with epigenetic dysregulation and associated changes in the expression levels of key genes related to neuronal and/or synaptic plasticity, including brain‐derived neurotrophic factor (BDNF) (Molinari et al., 2020), cAMP response element‐binding protein (CREB) (Yu et al., 2017) and sirtuin (SIRT‐1) (Herskovits & Guarente, 2014). Consistently, aged neurons presented a significant reduction in the transcriptional levels of BDNF, CREB, and SIRT1 (Figure 6). To understand if GPE could influence the transcription of the before mentioned genes, the cells were treated with GPE for 21 days and then a real‐time RT‐PCR was performed. GPE, at a concentration of 500 μM, induced a significant increase in the transcriptional levels of BDNF, CREB and SIRT‐1 (Figure 6a). These data suggest an activation of the intracellular pathways connected to neuronal plasticity. In parallel experiments, similar results were obtained incubating cells with choline alphoscerate (Figure 6a). Of note, choline alphoscerate induced significantly lower fold of increase in the transcription of the same genes than those elicited by GPE.

The protein expression of CREB, BDNF and SIRT1 was verified too by specific immunoenzymatic assays. Aged neurons presented significant minor levels of CREB and SIRT1, thus confirming the results evidenced at a transcriptional level. As depicted in Figure 6, GPE induced a significant enhancement in the expression of CREB and SIRT1; in contrast, no significant effect on BDNF levels was evidenced.

### 3.7 Evaluation of the expression of proteins related to neuronal plasticity: GSK‐3β

**FIGURE 7:**
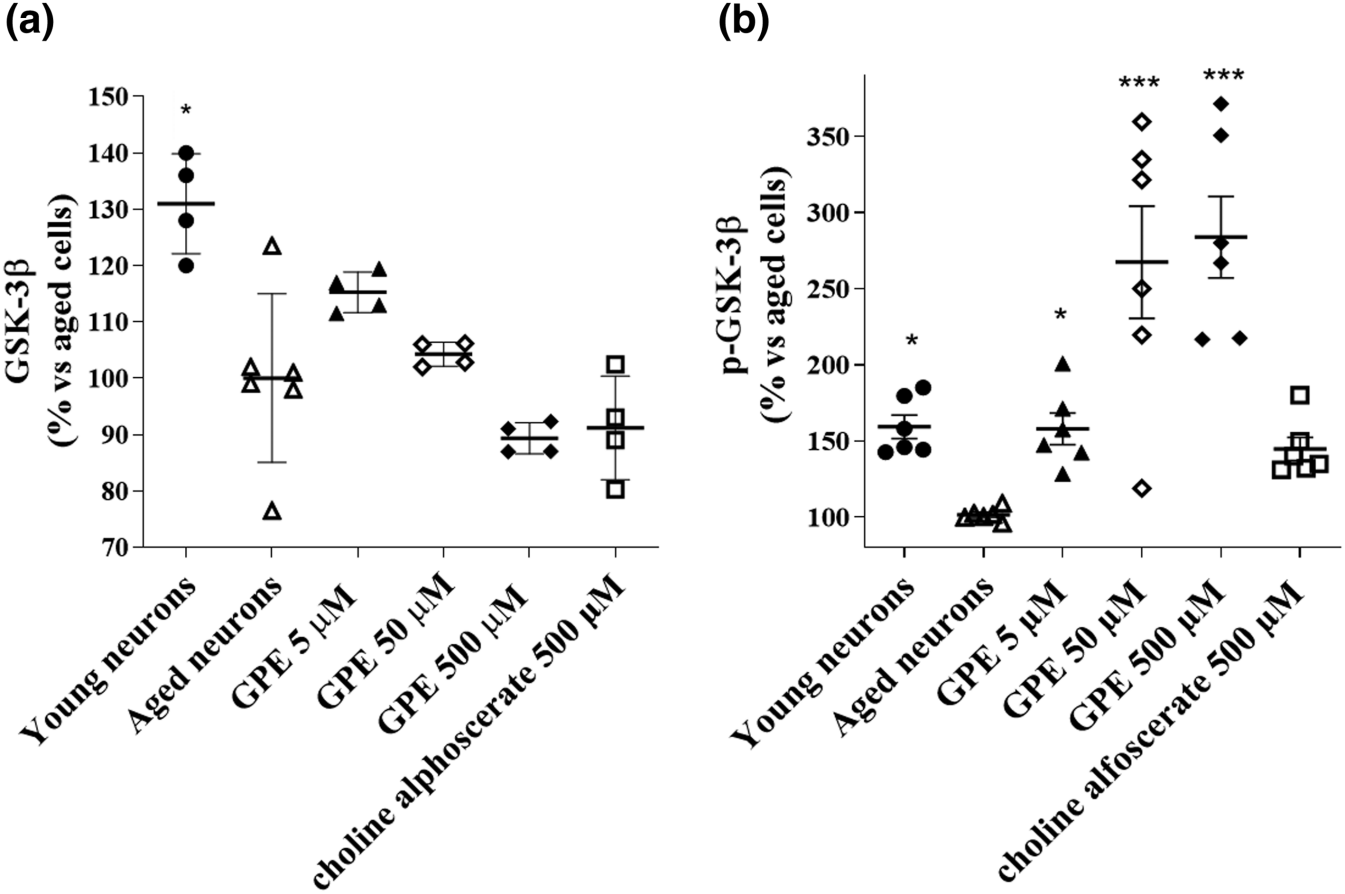
Evaluation of GSK‐3β expression. Aged cells were challenged with culture medium only (control cells) or GPE (5, 50 and 500 μM), or choline alphoscerate (500 μM) for 21 days. The expression of (a) total or (b) phosphorylated GSK‐3β was determined by specific enzyme immunoassays. The data are expressed as a percentage with respect to the aged control cells (set at 100%) and represent the mean ± SEM of three separate experiments, executed in triplicate. * *p* < 0.05, *** *p* < 0.001 versus aged cells

GSK3 is ubiquitously expressed in nerve cells, and it is involved in the regulation of both forms of synaptic plasticity, long‐term potentiation and long‐term depression, and in various neurological disorders (Salcedo‐Tello et al., 2011). Many studies confirm that GSK3 overactivation is the cause of Aβ protein accumulation, hyperphosphorylated forms of tau and inflammation that are related to the onset of neurodegenerative diseases (Salcedo‐Tello et al., 2011). However, aged cells did not have significantly higher levels of GSK3 than young neurons (Figure 7). GPE did not induce any significant changes in GSK‐3β levels compared to control cells (Figure 7). Similar results were obtained by incubating the cells with choline alphoscerate (Figure 7).

When the active phosphorylated form of GSK‐3**β** was examined, we noticed a significant decrease in aged neurons with respect to young cells (Figure 7). Nevertheless, GPE induced a significant enhancement of GSK‐3β phosphorylation, starting from 5 μM.

### 3.8 Evaluation of the protein levels associated with neurodegeneration

**FIGURE 8:**
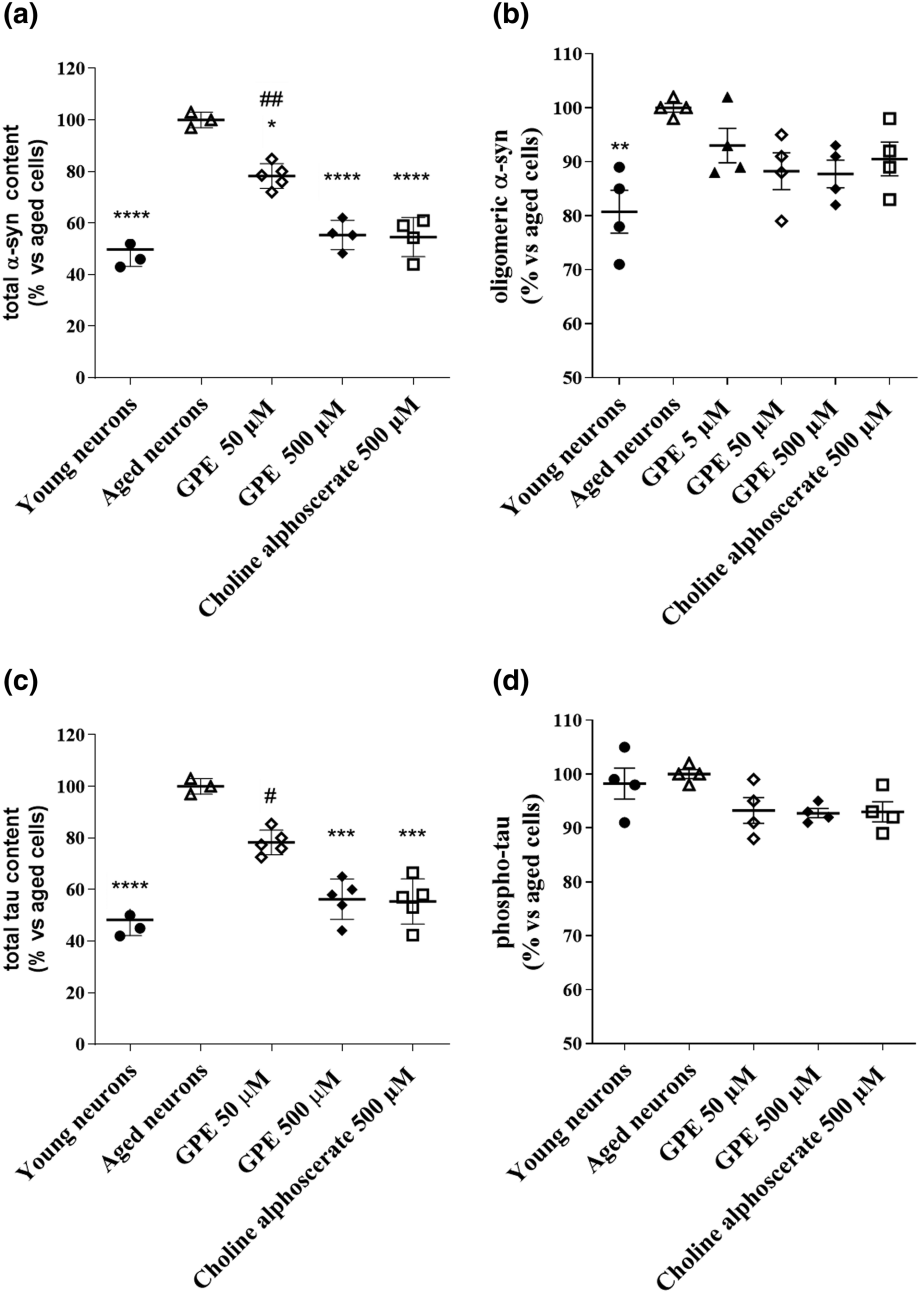
Evaluation of protein levels associated with neurodegeneration. Aged cells were challenged with culture medium only (control cells) or GPE (50 and 500 μM), or choline alphoscerate (500 μM) for 21 days. The content of (a) total or (b) oligomeric α‐synuclein, (c) total or (d) phosphorylated tau protein was determined by a specific enzyme immunoassay, as described in section 2. The data are expressed as a percentage with respect to the control (set at 100%) and represent the mean ± SEM of three separate experiments. *p < 0,05, ***p < 0,001, ****p < 0,0001 versus aged cells; #*p* < 0.05, ##*p* < 0.01 versus choline alphoscerate

Many studies highlight the beneficial activity of nootropic drugs or nutraceuticals against toxicity induced by an accumulation of misfolded proteins including tau and α‐synuclein (Alausa et al., 2021; Jia et al., 2011). Consistently, aged cells presented significantly higher levels of total and oligomeric α‐synuclein and protein tau (Figure 8). We then aimed to understand whether GPE can reduce the accumulation of misfolded proteins in ageing cells. As depicted in Figure 8a, GPE reduced the α‐synuclein accumulation, in a significant way starting from 50 μM. In parallel experiments, choline alphoscerate showed comparable reductions in the accumulation of α‐synuclein (Figure 8a). When the oligomeric form of the protein was considered, GPE reduced oligomeric α‐synuclein content in a concentration‐dependent manner, although this reduction did not reach statistical significance (Figure 8b). Similar results were evidenced for choline alphoscerate (Figure 8b).

Furthermore, GPE significantly reduced the tau protein content at 50 μM and 500 μM concentrations (Figure 8c). In parallel experiments, choline alphoscerate was able to decrease the accumulation of tau protein, with percentages comparable to those elicited by 500 μM GPE (Figure 8c). In contrast, no significant effect was evidenced on the phosphorylated form of protein tau, either by GPE or choline alphoscerate (Figure 8d).

## 4 DISCUSSION

Herein, GPE beneficial properties were investigated in an in vitro cellular model of ageing obtained by culturing human hippocampal neurons for 21 DIV. In this work, GPE was demonstrated to increase cellular metabolism by improving glucose uptake and complex I of the chain respiratory activity and of the GSK‐3β pathway. Moreover, GPE promoted cellular plasticity by inducing BDNF, CREB and SIRT1 gene and protein expression. Finally, GPE was demonstrated to reduce the accumulation of misfolded proteins related to ageing and neurodegeneration (i.e., α‐synuclein and tau). Overall, our data suggest that GPE can exert protective effects in a cellular model of ageing.

Ageing is characterised by a reduction in biological functions and cellular metabolism (Catic, 2018). In particular, the reduction of GLUT1 receptor levels and the impairment of mitochondrial activity have been widely demonstrated during physiological ageing (Castelli et al., 2019) The resulting reduction in ATP causes synapses alteration (Harris et al., 2012) that leads to memory loss, learning difficulties and attention deficit (Alexander et al., 2012; Dykiert et al., 2012). At the same time, the alteration of synaptic transmission strongly depends on the composition of the neuronal cell membrane which, during ageing, undergoes a significant change in its structure (Yehuda et al., 2002). A membrane low in fatty acids causes incorrect or decreased synaptic communication. For example, in many studies, a reduction in PE, phosphatidylinositol, PC levels have been found in people affected by Alzheimer disease (Prasad et al., 1998). Finally, some works have shown that low levels of PE can lead to accumulation of α‐synuclein (Wang et al., 2014) and that Aβ is regulated by cellular level of PE (Nesic et al., 2012). Herein, human hippocampal neurons were used to mimic an aged cellular model by culturing cells for 21 DIV, as previously reported (Daniele, Da Pozzo, et al., 2016; Daniele et al., 2020; Calvo‐Rodríguez et al., 2017). In our hands, aged cells presented a significant reduction in glucose uptake, oxidative phosphorylation and in the transcriptional levels of neurotrophic genes. Moreover, an accumulation of α‐synuclein and tau was evidenced too, thus validating the experimental model.

In the present paper, GPE effects on glucose metabolism, membrane plasticity and cellular well‐being were verified in aged human hippocampal neurons, which possess cholinergic circuits (Orta‐Salazar et al., 2014) and influence processes of learning and memory (Wood et al., 2000). Therefore, these cells can represent a good in vitro model to investigate nootropic properties of drugs. The induction of cellular ageing in vitro has been confirmed by the detection of common markers of DNA damage, that is, double strand break and phosphor‐ATM (Timchenko, 2020). Moreover, herein we noticed a progressive increase of p53 and p21 during cell culture up to 21 DIV. Consistently, literature has evinced a progressive p53 accumulation in aged mouse hippocampus (Bialuk et al., 2020) and in ageing cellular models (Wu & Prives, 2018) and increased levels of p21 triggering cell cycle arrest in senescent cells (Papismadov et al., 2017).

In this work, the cells were subjected to 21 DIV in vitro to mimic an ageing condition, and they were challenged with GPE or choline alphoscerate as a nootropic positive control, which is used for the improvement of cognitive dysfunction in patients with dementia of neurodegenerative and vascular origin as proven choline/acetylcholine precursor (Di Salvo, 2021; Parnetti et al., 2001; Perri et al., 1991).

Hippocampal cells at 21 DIV displayed a significant decrease in the amount of PLs, consistent with the age‐related changes in brain PLs that have been associated to cognitive dysfunction (Quan & Bakovic, 2021; Schverer et al., 2020). As expected, GPE counteracted the reduction in the PC and PE content, thus confirming that GPE promote PLs biosynthesis as evidenced in previous reports (Biggio et al., 2021; Daniele et al., 2020; Daniele, Da Pozzo, et al., 2016).

With the aim of investigating cellular metabolism, glucose uptake was evaluated in the ageing cellular model. GPE significantly increased glucose levels, with a maximal significant effect at 50 μM concentration, thus suggesting that the modulation of GLUT transporter by the nootropic compound is more sensible at lower doses; additional variables, including the time of treatment and the protein recycling, may influence this process. In parallel experiments, choline alphoscerate caused a significant increase in glucose uptake too, even if with lesser effects than GPE and at a higher concentration (500 μM) with respect to GPE, evidencing a less sensible modulation of the glucose transporter by this molecule.

Consistent with these data, it was shown that glucose transport activity of GLUT4 and GLUT3 is controlled by the membrane PL composition; in particular, PE and diacylglycerol have been demonstrated to increase glucose transport up to 3 times (Hresko et al., 2016).

Next, GPE effects on IGF‐1, which has been shown to stimulate glucose transport and play an important role in the processes of ageing and neuroinflammation, were investigated (Clemmons, 2004; Labandeira‐Garcia et al., 2017). Challenging cells with GPE or choline alphoscerate, no significant change in the levels of IGF‐1 was demonstrated. These results suggested that neither compound acted on the IGF‐1 pathway. Conflicting data have been reported in the literature. For example, long‐term change in PL composition has been shown to disrupt the IGF‐1 signalling pathway in C2C12 cells (Rauch & Loughna, 2005); in contrast, different studies have demonstrated that PE reduced insulin/IGF‐1‐like signalling in *Caenorhabditis elegans*. (Park et al., 2021).

The activity of respiratory chain complex I was evaluated to understand if the reduction in glucose utilisation can be associated to a lowered mitochondrial function. GPE significantly increased the activity of complex I, in a concentration‐dependent manner; in contrast, choline alphoscerate did not affect mitochondrial complex activity, thus suggesting that GPE may activate additional intracellular pathways than choline alphoscerate that can lead to an enhancement of mitochondrial functionality. Consistent with our data, a reduction of PE has been demonstrated to decrease respiratory capacity, ATP production and the activities of electron transport chain complexes (C) I and IV (Tasseva et al., 2013). Furthermore, an alteration in the PC metabolism has been related to a complex I dysfunction and an increase of the oxidative stress in the mitochondria of male Wistar rats (Hensley et al., 2000).

Numerous studies have shown that CREB, BDNF and SIRT1 are closely related to synaptic plasticity and play a crucial role in memory formation and cognitive functions (Budni et al., 2015; Michán et al., 2010; Silva, 2003). For this reason, the expression of these genes following GPE treatment was evaluated. The transcriptional levels of the above‐mentioned genes increased upon treatment with both nootropics, confirming their involvement in cellular well‐being. Similar results were obtained with choline alphoscerate, even with a lower extent. These data suggest that the two PL precursors possess different chemical structures related to slight differences in the restoration of intracellular pathways related to cellular ageing.

Immunoenzymatic assays confirmed that aged neurons presented significant lower levels CREB and SIRT1, consistent with the general decrease of sirtuins and neurotrophic factors reported in aged brain cells (Hattiangady et al., 2005; Herskovits & Guarente, 2014). GPE was able to counteract the decrease in the protein expression of CREB and SIRT1, thus confirming its neuroprotective action. Consistent with our data, EPA‐pPE and EPA‐PE alleviate cell death and improve neuronal cell morphology by activating the BDNF/TrkB/CREB pathway (Che et al., 2020). At the same time, cognitive deficits in SIRT1 knock‐out (KO) mice have been associated with defects in synaptic plasticity (Michán et al., 2010), and DHA‐PL and EPA‐PL have been shown to inhibit TNF‐α‐induced lipolysis in 3T3‐L1 adipocytes by activating the SIRT1 pathways (Yang et al., 2021).

The GSK3 plays a pivotal role in neuronal function, and its dysregulation has been related to the onset of different neurodegenerative and psychiatric disorders (Duda et al., 2018). Recent research has found that increased GSK3β activity is linked to the pathogenesis of AD through Aβ, phosphorylated tau and mitochondrial dysfunction (Reddy, 2013). For example, GSK3 inhibition has been demonstrated to improve the cognitive deficit in animal models of neurodegeneration (Griebel et al., 2019; Xing et al., 2016). Herein, total GSK3 expression in aged cells was not affected either following GPE or choline alphoscerate treatment. Nevertheless, when the active phosphorylated form of GSK‐3β was examined, we noticed a significant decrease in aged neurons with respect to young cells (Figure 7). Nevertheless, GPE induced a significant enhancement of GSK‐3β phosphorylation, starting from 5 μM. These data evidenced that the GPE‐cytoprotective effects in aged neurons involve the activation of GSK‐3β pathway.

As final step, GPE ability to counteract protein misfolded accumulation was investigated. GPE reduced both total and oligomeric α‐synuclein and total tau accumulation. In parallel experiments, choline alphoscerate showed comparable reductions in the levels of the analysed proteins. Consistent with this data, insufficient levels of PE cause α‐syn accumulation (Wang et al., 2014); furthermore, it has been shown that cell membrane composition could have an effect on the pathological aggregation of the tau protein (Bok et al., 2021).

## 5 CONCLUSION

In conclusion, the beneficial effects of GPE on glucose metabolism, on membrane plasticity and in counteracted misfolded protein accumulation were demonstrated in this work. Moreover, the drug increased cellular metabolism with improving glucose uptake and complex I of the chain respiratory activity and increased cellular well‐being through BDNF, CREB and SIRT1 transcription and protein expression. Furthermore, the GPE‐cytoprotective effects in aged neurons involve the activation of the GSK‐3β pathway. Finally, GPE was demonstrated to protect aged human hippocampal neurons from protein misfolded accumulation. Although choline alphoscerate is one of the most widely used inotropes, our results show that GPE possesses the best beneficial effects at the level of cellular metabolism and in inducing the expression of genes related to neuronal plasticity and cellular well‐being.

## CONFLICTS OF INTEREST

The authors declare no conflict of interest.

## AUTHOR CONTRIBUTIONS

E.Z. analysed the data and wrote the manuscript; S.D. performed experiments, analysed the data and revised the manuscript; L.C. performed experiments; C.M. acquired fundings and supervised the project; M.V. revised the manuscript; G.M. and L.R. contributed to project management, scientific contribution and manuscript revision. All authors have read and agree to the published version of the manuscript.

### PEER REVIEW

The peer review history for this article is available at https://publons.com/publon/10.1111/ejn.15783.

## Data Availability

The data presented in this study are available in the present article.
